# Comment on: “Intracytoplasmic morphologically selected sperm injection (IMSI) does not improve outcome in patients with two successive IVF-ICSI failures” by Gatimel et al.

**DOI:** 10.1007/s10815-016-0746-9

**Published:** 2016-05-30

**Authors:** K. Lukaszuk, E. Pastuszek, A. Samojedny

**Affiliations:** 1 INVICTA Fertility and Reproductive Center, Wroclaw Gdansk, Warsaw Poland; 2 Department of Obstetrics and Gynecological Nursing, Faculty of Health Sciences Medical, University of Gdansk, Gdansk, Poland

Dear Editor,

We have read with great interest the article “Intracytoplasmic morphologically selected sperm injection (IMSI) does not improve outcome in patients with two successive IVF-ICSI failures” by Gatimel and colleagues [1].

However, we have to bring attention to the problem which is unfortunately repeated in many articles and shows the weakness of our field in the application of basic physics. The authors state that the spermatozoa were observed under ×6000 magnification. It is a very misleading claim that makes it seem that it is indeed possible to obtain such magnification using a standard optical microscope (even a most advanced one). Calculation of the actual real magnification is impossible as the article does not list the basic microscope parameters—objective numeric aperture (NA), condenser NA and wavelength that was used to observe the spermatozoa [2, 3].

Nevertheless, let us try to calculate the actual real magnification used by the authors. We know that the objective’s magnification was ×100. It is described as an immersion microscope so we can assume the immersion was in oil (as opposed to air). The objective was mounted on the Leica DMI 6000 inverted microscope. Available objectives of this type have the NA from 1.3 to 1.49. Let’s assume the NA of 1.4.

Resolution, and consequently the real magnification, also depends on the condenser. As no specific information is provided, we could deduce that, in the best case scenario, the microscope had the condenser typically used in cell cultures and IVF laboratories. These kinds of condensers need to have a relatively large working distance that always means a lower condenser NA.

A condenser allowing moderate comfort of work that could be used with a ×100 objective would be TI-C-LWD LWD (numerical aperture 0.52).

Wavelength is another parameter that has not been provided. In order to get optimal resolution, a filer was probably used. We can assume the use of a standard filter, i.e., a green 550-nm filter. Resolution can be calculated based on the following formula:

$$ r=\frac{1.22\lambda }{N{A}_{\mathrm{obj}.}+N{A}_{\mathrm{cond}.}} $$

In a properly configured microscope: *NA*_obj._ + *NA*_cond._ = 2*NA*_obj._

We arrive at 0.35 μm. As the resolution of the human eye is approximately 300 dpi (dots per pixel) or 83 μm pixels on the screen 30 cm away from the human eye, we arrive at the magnification of ×237.

If we assume a much worse than usual eyesight (100 dpi) with the use of an ideal objective with NA of 1.49, T-C High NA condenser with NA of 0.85, and a 400-nm filter (blue filter that gives the highest resolution achievable with the human eye), we will get the real resolution of 0.21 μm and real magnification of only ×1199.

We can say, simplifying it greatly, that the real magnification of a microscope can be most easily estimated by multiplying the objective’s NA by 1000. It is a highly idealized model as exemplified by above calculations.

This should all be taken into account when information about microscopes is included in publications and when used to rationalize data and results.
